# A survey of women’s experiences of using period tracker applications: Attitudes, ovulation prediction and how the accuracy of the app in predicting period start dates affects their feelings and behaviours

**DOI:** 10.1177/17455057221095246

**Published:** 2022-04-25

**Authors:** Anna Broad, Rina Biswakarma, Joyce C Harper

**Affiliations:** 1 EGA Institute for Women’s Health, University College London, London, UK; 2 Institute for Education, University College London, London, UK

**Keywords:** menstrual cycle, ovulation, period app, period tracker, period

## Abstract

**Introduction:**

Using an online survey, the aim of this study was to ask women about their real-life experiences of using period tracker apps, their attitudes towards using their app, the information the app provided regarding ovulation and how the accuracy of the app in predicting period start dates affects their feelings and behaviours if their period comes earlier or later than predicted.

**Methods:**

This mixed-methods observational study was conducted by an online survey of 50 multiple-choice and open-ended questions. The survey was generated with Qualtrics XM^®^ and promoted via social media. It was open to any person who had used a period tracker.

**Results:**

From 375 total responses, 330 complete responses were obtained, giving a completion rate of 88.0%. Respondents were aged between 14 and 54, with a mean age of 26.0 (±7.81). When asked what was the best thing about using the app, 29.7% (98/330) of respondents selected ‘To know when I’m ovulating’. Respondents were asked if their period ever started earlier than the app predicted; 54.9% (189/330) said it had and 72.1% (238/330) said it had started later than predicted. When asked how they felt if their period arrived earlier or later than expected, thematic analysis of periods starting earlier revealed four themes: feeling unaffected, being frustrated/unprepared, feeling anxious/stressed and feeling confused/intrigued. Thematic analysis when their period arrived later revealed six themes: anxious/concerned about pregnancy, disappointed about pregnancy, seeking advice/informing healthcare professionals, thoughts about menopause, feeling unaffected and being better prepared.

**Conclusion:**

Period trackers need to be clearer on their intended use and reliability, especially for period due date and ovulation. Qualitative analysis shows the impact of inaccurate predictions on aspects of the users’ health. This study calls for period tracker app companies to update their apps to provide transparency to their users about their intended use and capabilities.

## Introduction

FemTech (female-focused technology designed to aid in women’s health) apps range from fertility-based apps for pregnancy planning and contraception, to menstrual cycle apps for the tracking of periods and symptoms. They are a popular and ever-developing field, with more than 200 million downloads.^1^ The apps that allow the monitoring of menstrual cycles, commonly known as period tracker apps, are the fourth most popular health app among adults.^2^ Since the release of the first-ever period tracker, Glow, in 2013, the industry has continuously expanded, with the FemTech market estimated to be worth $50 billion by 2025.^3^

Fertility-based FemTech apps are marketed towards women wanting to know when they should or should not be having sexual intercourse to achieve or avoid pregnancy.^4^ They are able to do this through user input of biological markers, described as fertility awareness–based methods (FABM), which predict ovulation. These markers include oral basal body temperature, cervical mucous consistency and urinary LH levels, which all increase at, or prior to, ovulation.^4^ However, generally, fertility apps use a calendar-based algorithm to predict ovulation, which has been shown by various authors to be ineffective in predicting ovulation.^1,5^

Today, the choice of period tracker apps is vast, with 7% of the 90,088 health apps in the Apple store focussed on women’s health and pregnancy.^2^ However, it is important that the app a woman chooses to download provides accurate information, is effective in predicting her period and is beneficial to her lifestyle. Period tracker apps have a responsibility to users as incorrect information about the menstrual cycle could cause stress when periods are earlier or later than expected, and giving incorrect information about ovulation could lead to pregnancy or infertility.

There have been several studies examining women’s experience of using period tracker apps using surveys and interviews done in Austria and Spain,^6^ the USA,^7^ Australia^8^ and New Zealand.^9^ Women report using the apps to plan and prepare for upcoming periods; verify menstrual cycle experiences; inform healthcare professionals (HCPs) confidently and plan pregnancy and contraception. But negative effects of using period tracker apps have been reported in these studies, including reports of strong emotional effects when the app presented a period start date that differed from the actual start date.^6^ This is of concern when a report regarding the quality and effectiveness of period tracker apps found many of the apps which claim to be ‘evidence-based’ were not tested in trials whatsoever.^10^ This is supported by Zwingerman et al.,^11^ whose appraisal of menstrual tracking apps showed 22.1% of apps to contain serious inaccuracies. A study by Worsfold et al.^12^ analyzed the ability of period tracker apps to accurately predict the length and dates of five different cycle profiles representing a range of real-life cycles. They found variation between apps in their prediction of fertile days, day of ovulation and period start dates. They concluded calendar-based apps that predicted ovulation and the fertile window were giving inaccurate information. This highlights the inaccuracies and potentially serious consequences of period tracker apps.

In the UK, regulatory efforts are being put in place by bodies with the launch of the National Health Service (NHS) Health Apps Library and the National Institute for Health Care and Excellence (NICE)^13^ recently releasing an Evidence Standards Framework to which health apps must comply. However, a recent study concluded that most apps did not have sufficient evidence to meet the minimum criteria of the NICE’s framework and that apps included in the NHS Health Apps Library presented no more evidence than apps only found in the Apple app store.^14^ This conveys the need, and also the challenges, of regulating and keeping up with a constantly updating field of technology.

The aim of this study was to ask women about their real-life experiences of using period tracker apps, their attitudes towards using their app, the information the app provided regarding ovulation and how the accuracy of the app in predicting period start dates affected their feelings and behaviours. It is hoped that the results of this survey will add to the literature highlighting the benefits and risks of such apps.

## Materials and methods

### Ethics

This research was approved by University College London (UCL) Research Ethics Committee ID Number: 9831/004, with no anticipated risks for the participants. The first question of the survey was to agree to informed consent. All data were collected and have been presented anonymously and managed in accordance with the Data Protection Act of 1998.

### Participants

This observational mixed-methods study distributed an online survey to people who use, or have used, a period tracker app. The inclusion criteria were any woman who had used a period tracker. There were no specific exclusion criteria. There was no age limit on respondents to gain an inclusive and representative insight of all users’ experiences. Respondents were asked to confirm if they use, or have ever used, a period tracker app.

### Materials

A 50-item survey was developed, guided by preliminary polls and discussions launched via social media. The questions were designed to address demographics, menstrual cycle characteristics and period tracker app use to ensure the collection of relevant data. The phrasing of the survey was formulated to avoid irrelevant and leading questions, and participants were always offered a ‘Prefer not to say’ option. Respondents had to answer each question but it was possible for the respondents to stop answering questions and submit the survey, hence some incomplete responses. The survey was in English, with simple and focussed questions to allow the participation of as many users as possible. Qualtrics XM^®^, a mass distribution and data analysis tool, was used to design, promote and collect the results of the survey. The survey is available in supplementary data 1.

Before circulation, validation of the final survey took place through cognitive interviewing sessions. During these pilot responses, participants were interviewed as they went through the survey to ensure its ease of use and the correct understanding of each question. To further confirm this, the survey was first distributed among a select group run by Joyce Harper (www.globalwomenconnected.com via Facebook), prior to mass online distribution on social media via Joyce Harper and Anna Broad’s Instagram, Twitter, LinkedIn and Facebook pages. The survey was live for 19 days, from 30 June to 20 July 2021.

### Data analysis

Due to the large data set, this article analyzes the findings of a sub-set of data, with a second paper in preparation. This article focuses on demographics and menstrual cycle characteristics, as well as the questions regarding respondents’ use of period trackers, their attitudes to inputting data, the influence of the apps on sexual intercourse, ovulation prediction and the effect of the app’s predicted period start dates on respondents’ feelings and behaviours.

Inductive thematic analysis was used for the qualitative data.^15^ Two questions were analyzed: how they felt when their period started earlier than the app’s predicted start date and how they felt when their period started later than the app’s predicted start date. The responses were read and then re-read to enable immersion into the text and to identify meaningful and repeated units of text. These units of text were assigned codes, allowing more data to be identified and categorized into these codes. The data were reviewed again to ensure all codes had been identified and exhausted, following which the codes were grouped into themes.

## Results

A total of 375 surveys were started and 330 completed the survey giving a completion rate of 88.0%.

All figures show the percentage of responses to each question. For some questions, respondents were asked to select all that apply or three answers that apply, which are detailed in the figures.

### Respondent demographics

Table 1 shows the full details of the demographics, highlighting 91.5% (302/330) of respondents were from the UK. The average age was 26.0 and the majority of respondents described themselves as White British, 73.3% (242/330), and heterosexual, 87.0% (287/330). Most of the respondents were in a relationship, 66.0% (218/330), either married or in a civil partnership, co-habiting, or not co-habiting.

**Table 1.** Detailed demographics of all respondents.

Demographics	Mean (SD)
Age	26.0 (7.81)
Country of residence	Frequency (%)
UK	302 (91.5)
Other	28 (8.5)
Ethnicity	
White-English/Welsh/Scottish/Northern Irish/British	242 (73.3)
White-Irish	12 (3.6)
Any other White background	35 (10.6)
Black/Black-British – African	6 (1.8)
Black/Black-British – Caribbean	3(0.9)
Asian/Asian-British – Indian	10 (3.0)
Any other Asian background	9 (2.7)
Latino	4 (1.2)
Arab	2 (0.6)
Mixed ethnic background	14 (4.2)
Other	2 (0.6)
Prefer not to say	1 (0.3)
Sexual orientation	
Heterosexual	287 (87.0)
Homosexual	3 (0.9)
Bisexual	30 (9.1)
Pansexual	5 (1.5)
Asexual	2 (0.6)
Prefer not to say	2 (0.9)
Educational level	
Secondary school	5 (1.5)
A-level/college level	62 (18.8)
University undergraduate	140 (42.2)
University postgraduate	115 (34.9)
Other	8 (2.4)
Prefer not to say	0 (0)
Relationship status	
Single	109 (33.0)
In a relationship not co-habiting	96 (29.1)
In a relationship co-habiting	66 (20.0)
Married/civil partnership	56 (17.0)
Other	0 (0)
Prefer not to say	3 (0.9)
Religion or belief	
No religion or belief	198 (60.0)
Christian	105 (31.8)
Hindu	8 (2.4)
Jewish	1 (0.3)
Muslim	9 (2.7)
Sikh	0 (0)
Buddhist	0 (0)
Other	3 (0.9)
Prefer not to say	6 (1.8)
Disability status	
No disability	289 (87.6)
Specific learning difficulty or disability	16 (4.8)
Long-term illness or health condition	18 (5.5)
Sensory impaired	4 (1.2)
Physical or mobility impaired	3 (0.9)
General learning disability	0
Autistic spectrum disorder	2 (0.6)
Other	2 (0.6)
Prefer not to say	2 (0.6)

The respondents were highly educated, with 77.1% stating their highest educational qualification to be either an undergraduate degree, 42.2% (140/330), or postgraduate degree, 34.9% (115/330).

A large proportion of respondents described themselves as having no religion or belief, 60.0% (198/330), or being Christian, 31.8% (105/330). Respondents mainly had no disabilities, 87.6% (289/330).

### Menstrual cycle characteristics

**Figure 1.**
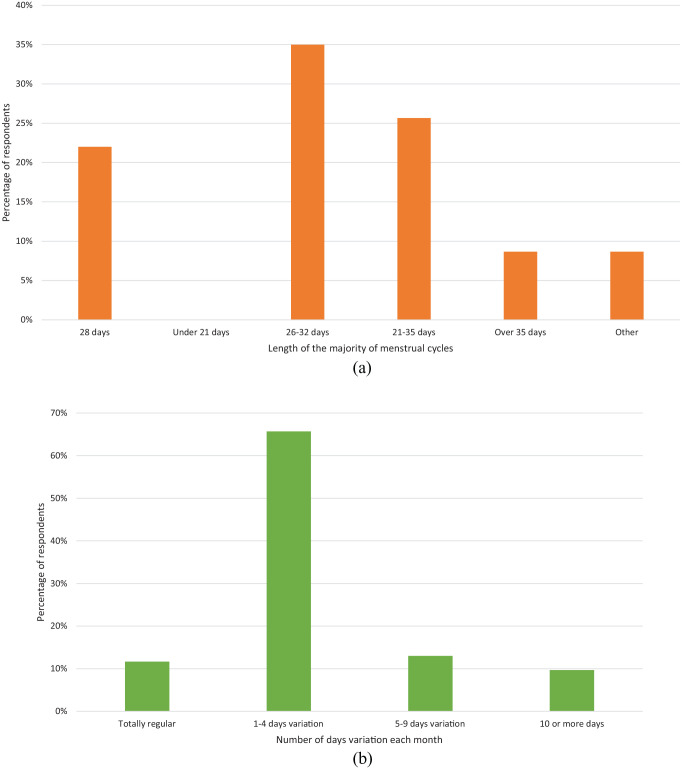
A graph showing the women’s responses regarding the characteristics of their menstrual cycle (a) Menstrual cycle length and (b) variation in respondents’ cycle length each month. Of 300 women, most showed a 26- to 32-day cycle with 1–4 days variation in cycle length.

There were several questions that provided insights into the respondents’ menstrual cycle characteristics, one of which 10 of 300 women answered as not currently having periods. Most respondents described their menstrual cycle to be between 26 and 32 days, 35.0% (105/300), (Figure 1(a)) and that it tended to vary by 1–4 days each month, 65.7% (197/300) (Figure 1(b)).

**Figure 2.**
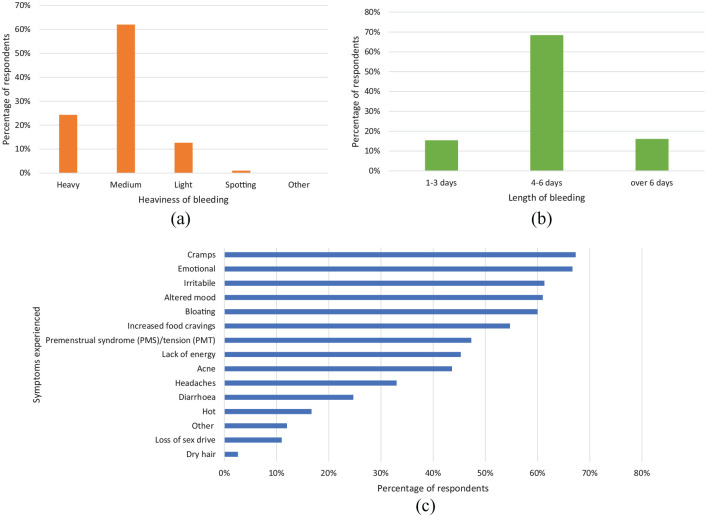
A graph showing the women’s responses to the characteristics of their menstrual cycle bleed (a) heaviness of bleeding (b) length of bleeding and (c) the different symptoms experience when their period is due. Of 300 women, the majority defined their bleeding as medium, lasting for 4–6 days and reported a variety of symptoms when their period was due. For Figure 2(c), the respondents were able to tick all the answers that applied.

When asked about period length and heaviness of bleeding, most respondents described their periods to be medium, 62.0% (186/300), and 4–6 days in length, 68.5% (204/300) (Figure 2(a) and (b)). Respondents were also asked about the symptoms they experience in the days before their period is due and could select all that applied. Figure 2(c) shows respondents had a wide range of symptoms, with the most common being cramps, 67.3% (202/300).

### The use of period tracker apps

**Figure 3.**
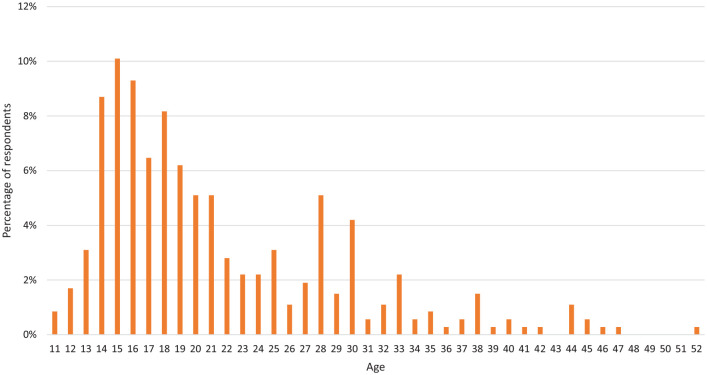
The age at which respondents began using a period tracker app. The graph shows that the majority of respondents started using the app when they were teenagers.

Respondents were asked questions to discover and understand their use of period trackers. The mean age of first downloading a period tracker app was 21.4 (±7.86), with a minimum age of 11 and a maximum age of 52 (Figure 3).

**Figure 4.**
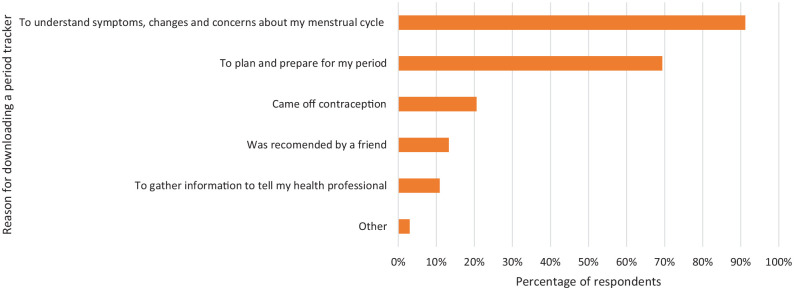
The reasons why respondents downloaded and started using a period tracker app. They were able to tick all the answers that applied. Of 330 women, the major reason was to understand their symptoms, changes and concerns about their menstrual cycle.

Respondents were also asked about the reasons why they first started using a period tracker. Most respondents wanted to understand their symptoms, changes and concerns about their menstrual cycle, 91.2% (301/330) and to prepare for their period, 69.4% (229/330) (Figure 4).

### The best thing about using a period tracker

**Figure 5.**
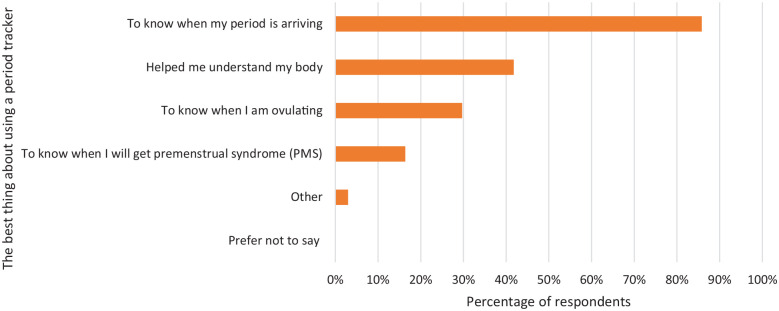
A graph showing the women’s responses to the question ‘what is the best thing about using a period tracker app?’. They could tick all options that applied. From 330 responses, the majority said ‘to know when my period is arriving’.

The respondents were asked what they felt was the best thing about using a period tracker (Figure 5). The first and second most commonly selected statements were ‘To know when my period is arriving’, 85.8% (283/330), and ‘Helped me understand my body’, 41.8% (138/330). However, 29.7% (98/330) of respondents selected ‘To know when I’m ovulating’.

### The influence of period trackers on sexual intercourse

The majority of respondents’ sexual activity was not influenced by their app’s predicted dates, 65.5% (216/330). However, a total of 17.0% (56/330) of respondents stated that it was, with 10.9% (36/330) of all respondents stating they avoid having sex on the fertile days predicted by the app.

### Respondents’ attitudes towards entering data

When respondents were asked to select the phrase that best described their attitude towards entering data into their app, the two most common statements were ‘Entering my data is part of my routine’, 43.0% (142/330), and ‘I often forget to enter my data as it is not a priority’, 37.0% (122/330).

Questions regarding privacy concerns when using a period tracker showed an overall majority, 83.0% (274/330), of respondents had no privacy concerns. When asked to further explain their attitudes towards data privacy, many stated it either had not even crossed their mind, or that they feel that as they have grown up with the age of the Internet, access to personal data is expected.

### Extent of predicted start date accuracy

**Figure 6.**
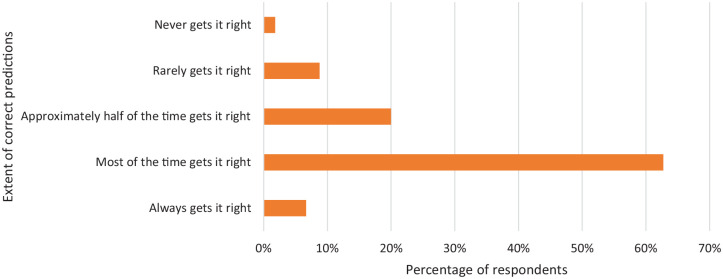
A graph showing the women’s responses to being asked the extent to which period tracker apps accurately predict the start date of their period. The majority of respondents said that their period tracker got it right most of the time.

Figure 6 shows the majority said their app gets their period start date right most of the time, 62.7% (207/330), while 6.7% (22/330) said their app predicts it correctly all of the time. However, 8.8% (29/330) said the app rarely gets it right.

Respondents were asked if their period ever started earlier than the app predicted; 54.9% (189/330) said it had, 24.9% (82/330) said it never had and 20.3% (67/330) said sometimes. When asked if their period ever started later than the app predicted; 72.1% (238/330) said it had, 10.6% (35/330) said it never had and 17.3% (57/330) said sometimes.

### The effect of a period earlier than predicted

There was an opportunity for respondents to write a free text about how they felt when their period started earlier than the app’s predicted start date. If respondents had nothing more to say, they could state ‘None’. Overall, there were some contrasting responses, ranging from feeling as though it did not really affect them, to strong feelings of anxiety, frustration and confusion. Thematic analysis of the 145 detailed responses highlighted four key themes: feeling unaffected, being frustrated and unprepared, feeling anxious and stressed, and feeling confused and intrigued.

### Feeling unaffected

A large number of respondents described how their period starting earlier than the app’s predicted start date was not a surprise and it did not bother them. Some even stated that this was because they did not trust or believe the predictions.

*‘Coming early didn’t worry me’ (Aged 21, in a relationship co-habiting)*

*‘I don’t mind because I know my period will be around that time so I am not fussed’ (Aged 23, in a relationship not co-habiting)*

*‘Just one of those things’ (Aged 31, in a relationship co-habiting)*

*‘Don’t believe the predictions anyway so was amused’ (Aged 49, married/civil partnership)*

Reasons for feeling unaffected by an earlier-than-predicted period tended to fall under two sub-themes. First, respondents explained how they understand their body and know their menstrual cycles are irregular; and that they understand the signs and symptoms of their period.

*‘I can nearly always tell the day I will start my period because there is a significant drop in my basal body temperature’ (Aged 24, single)*

*‘I go by my symptoms as that’s when I know my period is going to start but just use the app as a rough guide’ (Aged 23, in a relationship co-habiting)*

*‘I didn’t think much of it as my periods have always been irregular and unpredictable due to the PCOS’ (Aged 24, in a relationship not co-habiting)*

*‘My periods can be erratic so was not bothered’ (Aged 46, married/civil partnership)*

On the other hand, many other respondents felt unaffected because they never expected the app to be 100% accurate.

*‘I felt fine – the app predicts it the best it can but I understand my body doesn’t run according to an app’ (Aged 25, in a relationship cohabiting)*

*‘I generally took the app’s predictions as just that – predictions not facts, so it didn’t bother me’ (Aged 22, in a relationship not cohabiting)*

However, in some cases, this lack of expectation of the app’s accuracy led respondents to blame their own body and periods when looking for a reason to understand what is presented by the app as an early period. This sub-theme appeared across varying ages and relationship statuses.

*‘I don’t especially blame the app, it’s more directed at my own body’ (Aged 38, married/civil partnership)*

*‘Frustrated at own period and its irregularity’ (Aged 23, in a relationship cohabiting)*

*‘I never felt it was the apps fault, more that my period was just irregular’ (Aged 18, single)*

### Feeling frustrated and unprepared

Another frequently mentioned feeling about having an earlier-than-predicted period was a sense of being surprised, annoyed and unprepared. Respondents mentioned how this inaccuracy meant they did not have access to suitable period products and that they would have worn different clothing. These feelings of frustration made some respondents feel upset, particularly if individuals greatly suffer while on their period, due to disorders such as endometriosis.

*‘I was unprepared with sanitary products which was inconvenient’ (Aged 42, married/civil partnership)*

*‘Annoyed because I didn’t have the right underwear on’ (Aged 23, in a relationship cohabiting)*

*‘Slightly underprepared – for instance I had no tampons at home as I thought I had time to buy them before I started’ (Aged 22, in a relationship cohabiting)*

*‘Upset. I get severe pain because of endometriosis so I try to plan ahead by not being at work if possible, close to painkillers, my heat belt and peace and quiet’ (Aged 30, single)*

The feelings of frustration commonly appeared with regards to events such as holidays, or other activities including exercise, that respondents had planned to take place around their period due dates.

*‘Frustrated-it can mean that I’ve planned specific events to avoid my period, then my period happens anyway’ (Aged 38, married/civil partnership)*

*‘I just need to know more or less when it will be, but it does help me to plan my activities (e.g. travel, sports)’ (Aged 33, in a relationship cohabiting)*

### Feeling anxious and stressed

Many respondents wrote how they felt worried, stressed, and even let down when their period came earlier than their tracker predicted. Respondents that were left feeling anxious described how this was in relation to pregnancy and ovulation dates, either because they were trying to conceive or trying not to.

*‘Disappointed as trying to conceive’ (Aged 33, in a relationship cohabiting)*

*‘Went in to check whether I had noted my ovulation date to be earlier than the app had predicted’ (Aged 44, married/civil partnership)*

*‘Worried ovulation day was also wrong’ (Aged 32, single)*

In certain cases, for those respondents who may have believed they were pregnant, an earlier-than-predicted period caused stress surrounding implantation bleeds.

*‘Would make me think it was possibly an implantation bleed, became stressful’ (Aged 35, in a relationship cohabiting)*

### Confused and intrigued

A final prominent theme in this analysis was that, although respondents may have been confused as to why their period was early, they were also intrigued and wanted to find out why this could have happened. This was particularly mentioned when the predicted start date was wrong by weeks, rather than days.

*‘Wondered why my cycle was so much shorter than usual’ (Aged 30, in a relationship cohabiting)*

*‘If it’s a week or more early, I think about what could have caused it’ (Aged 21, single)*

### The effect of a period later than predicted

Respondents were asked how it made them feel when their period was later than the apps predicted start date. This was also an open-ended question and respondents had the opportunity to write ‘None’ if they felt they had nothing more to say. Although this set of responses still showed some contrasting feelings, a much greater proportion of respondents described a sense of anxiety and panic that they could be pregnant. A late period also appeared to uncover worries over other reproductive health anxieties, including menopause, causing respondents to seek medical advice. After thematic analysis of the 175 responses given, six themes emerged: anxious and concerned about being pregnant, disappointed about not being pregnant, seeking advice and informing HCPs, thoughts about menopause, feeling unaffected and being better prepared.

### Anxious and concerned about being pregnant

One of the most frequently cited themes was the worry and fear about an unplanned pregnancy when respondents’ periods were later than predicted. Some mentioned how even though they knew it was likely to be part of their normal variation in cycle length, a late period still made them feel worried about pregnancy.

*‘Anxious that I might be pregnant’ (Aged 28, married/civil partnership)*

*‘I’m worried about pregnancy, especially if I’ve had sex recently. Even though I know it’s normal for me to be late, it still makes me worried*’. *(Aged 24, in a relationship not cohabiting)*

*‘panicked because the automatic assumption is then that you’re pregnant*’. *(Aged 20, in a relationship not cohabiting)*

*‘Slightly panicked as it had always been very reliable. I’ve completed my family now and have no desire for any more children so it was a bit worrying that it hadn’t started*’. *(Aged 35, married/civil partnership)*

*‘Anxious because I was just waiting for it to come, and worried about being pregnant*’. *(Aged 20, in a relationship not cohabiting)*

Further to this, respondents were aware that anxiety can delay periods further. They described how the nerves and stress created by a late period, as classified by their period tracker, can cause their period to start even later.

*‘Worried as I stress about it, which can delay it even more*’. *(Aged 22, in a relationship not cohabiting)*

*‘I was very anxious and i think the nerves kept delaying my period further*’. *(Aged 23, single)*

However, an exception to this theme mentioned by a few respondents was that because they were not sexually active at the time, they were not worried about an unplanned pregnancy. Still, a few highlighted how a later-than-predicted period made them worried about other aspects of their health, including general health.

*‘When I used a tracker I wasn’t sexually active so being a couple days late never bothered me, but if I was to be using one now I am sexually active it would bother me because of the pregnancy panic*’. *(Aged 22, in a relationship not cohabiting)*

*‘A bit worried/stressed. . .not worried about pregnancy just about general health’ (Aged 21, single)*

### Disappointed about not being pregnant

On the other hand, if a respondent was trying to conceive and pregnancy was what they were hoping for, respondents’ attitudes towards a late period appeared more hopeful and excited. For some, it seemed that they did not want to get their hopes up, and if their late period eventually started, respondents described a sense of disappointment.

*‘Worried I may be pregnant. Possibly secretly excited. happy*’. *(Aged 37, married/civil partnership)*

*‘Hopeful at the time!! Then upset when it came*’. *(Aged 32, married/civil partnership)*

### Seeking advice and informing HCPs

Many respondents felt the need to speak to their doctor when their period was late. Respondents described how as they became concerned and worried over their cycle length or delayed period, they looked for advice from their General Practitioner (GP).

*‘I also find it concerning (yet important) to see when my cycles abnormally long, and have phoned my GP for medical advice’ (Aged 24, in a relationship not cohabiting)*

*‘It lets me see I’m still within a non-worrying cycle length and gives info for doctor if required (I’m not sexually active so this would likely be evidence of a health issue)’ (Aged 37, single)*

Discussing the apps that predicted period dates with HCP became significant to respondents and HCPs when trying to understand if something was wrong and to reach a diagnosis.

*‘Frustrating as doctors overlook my condition due to my age and seeing “60 days late” for period is disheartening’ (Aged 21, in a relationship cohabiting)*

*‘The data helped me get diagnosed with PCOS*’. *(Aged 21, in a relationship not cohabiting)*

### Thoughts about the menopause

A theme most likely to appear within responses from older respondents was how a late period caused them to begin thinking about whether they were becoming peri-menopausal.

*‘Thoughts about the menopause’ (Aged 54, in a relationship not cohabiting)*

### Feeling unaffected

In contrast to the previous theme, those who were already aware that they were menopausal were not that affected by their period trackers prediction.

*‘Not surprised as menopausal – though found it funny that it bothered to predict when cycles so clearly unpredictable’ (Aged 49, married/civil partnership)*

Others had similar views and feelings as to when their period was earlier than predicted. Many respondents wrote analogous responses, mentioning again how they understood their body does not run according to an app and that they knew their cycle can be irregular, so it was to be expected.

*‘Again, I didn’t really think of it in relation to the app. I’ve always presumed the app works out my average cycle length and goes with that. I find it useful to know vaguely when my period is starting and use it for that only. My cycles aren’t totally regular to the day*’. *(Aged 27, in a relationship cohabiting)*

*‘I know that I have health factors that impact my period so it doesn’t worry me’ (Aged 21, in a relationship not cohabiting)*

*‘Again, I know I’m not regular so I wasn’t particularly worried’ (Aged 22, in a relationship not cohabiting)*

Some respondents described how their use of contraception allowed them to not feel worried or anxious, and that a late period was more of an inconvenience.

*‘I’m on the pill so I wasn’t too worried about it. It was more of an inconvenience than stressful*’. *(Aged 25, in a relationship not cohabiting)*

*“A bit stressed but otherwise fine because I trusted the contraception I’d used more than the app. It made me less willing to trust the app, however. (Aged 18, in a relationship not cohabiting)*

### Being better prepared

A positive theme that became apparent was that, compared to an earlier-than-predicted period, respondents felt that they were better prepared. Responses showed how a period later than predicted allowed access to suitable period products; some described how they even prefer a later than predicted period to earlier than predicted.

*‘if it’s a couple of days late then that’s fine because I’ve prepared for it’ (Aged 25, in a relationship cohabiting)*

*‘Was only by a few days and I was able to prepare*’. *(Aged 21, single)*

*‘I find that later is less annoying than earlier as you are still prepared for it, so I didn’t mind too much*’. *(Aged 19, in a relationship not cohabiting)*

However, one individual’s comment on this theme mentioned how having a late period meant that tampons and period pads were unnecessarily used.

*‘I know my cycle is a little irregular but it’s nice to be prepared. I do hate the wasted tampons and pads though*’. *(Aged 20, in a relationship not cohabiting)*

### Overall feelings about using a period tracker

**Figure 7.**
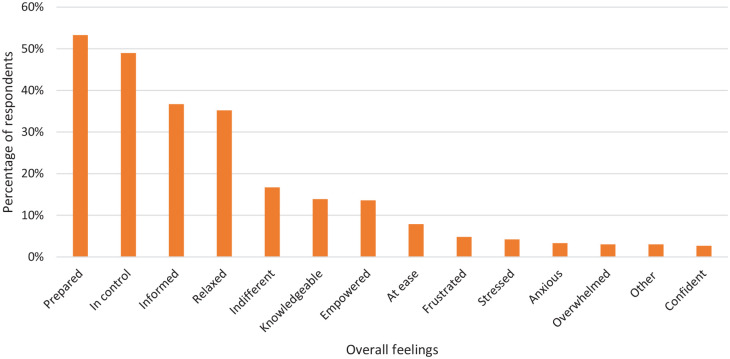
A graph showing the respondents’ overall feelings about using a period tracker app. They were able to pick three answers. The majority of feelings chosen were positive.

The final part of the survey asked respondents to select up to three feelings that describe how using a period tracker makes them feel (Figure 7). Overall, the majority of respondents expressed a positive experience; the top three feelings selected were ‘Prepared’, 53.3% (176/330), ‘In control’, 49.0% (162/330) and ‘Informed’ 36.7% (121/330). ‘Indifferent’ was commonly selected, 16.7% (55/330), while the most common negative feeling selected was ‘Frustrated’, 4.8% (16/330). There was a range in feelings towards using a period tracker.

## Discussion

Using an online survey, the aim of this study was to ask women about their real-life experiences of using period tracker apps. Specifically, their attitudes towards using their app, ovulation prediction and how the accuracy of the app in predicting period start dates affects their feelings and behaviours if their period comes earlier or later than predicted by the app. This is essential to ensure app-facilitated period tracking is fit for purpose. While some studies have been done using interviews and surveys in other countries, this was the first UK-based survey.

Results highlighted the range in users’ ages, careers and stages of life, as well as the range in attitudes towards entering data. Further findings showed a proportion of respondents would either have or avoid having sexual intercourse on their potential fertile days. Interestingly, most respondents had no concerns regarding data privacy when using the apps. Only a small proportion of respondents stated their app always predict their period correctly, and inaccurate predictions were more likely to result in a period later than predicted. The qualitative analysis further confirmed the range of the apps’ effects on women’s feelings and behaviours, although a period later than predicted tended to have a greater negative effect than earlier than predicted periods.

### Characteristics of period tracker users

The most frequently selected cycle length of 26–32 days corresponds with a recent study that investigated over 600,000 menstrual cycles, which found a mean cycle length of 29.3 days.^1^ Further consistent findings of cycle length were found by Faust et al.,^16^ who reported a mean cycle length among their 225,596 cycles to be 29.6 days. This study found 64.1% of respondents to have a variation of 1–4 days, while Grieger and Norman,^17^ who looked into menstrual cycle patterns of users of the app Flo, found 69% of participants to have <6 days variation. When comparing the heaviness of respondents’ periods with previously reported findings, fewer data are available. While a 2004 study measured blood loss in those referred with potential menorrhagia, the lack of population-wide studies investigating period heaviness makes it difficult to put current findings into context.^18^ This study found that 61.7% of respondents described the heaviness of their period to be medium and 67.9% described their period to last 4–6 days. The latter finding is consistent with a study carried out by the World Health Organization (WHO), which found a mean period length of 4.7 days; however, the date and the early post-menarcheal nature of this study’s sample must be considered.^19^

### Use of period trackers

Results of the survey showed an age range of 40 years between respondents. Although the mean age was 26.0, this broad range highlights the role period tracker apps play at every stage of a women’s reproductive life. The increase in downloading apps around the age of 14–16 is likely to align with the onset of menses, whereas the increase around the age of 28–30 could align with the desire to start a family. The most recent data shows the average maternal age for first children in the UK to be 28.9 years and could therefore be suggested that there is an increase in period tracking at this age as women start to plan to conceive.^20^

The reasons why women downloaded a period tracker varied. ‘Wanting to plan and prepare for my period’ was the most frequently selected response and ‘Coming off contraception’ included those trying to become pregnant and those coming off hormonal contraception. However, four specific categories about their own menstrual cycle (‘Wanted to know more about my menstrual cycle’, ‘Was worried about my menstrual cycle’, ‘To understand changes in menstrual cycle symptoms such as mood’ and ‘Suffering from PMS’), contributed to an overall 91.2% of users downloading a period tracker because they wanted to understand more about their body and menstrual cycle. This idea of women wanting to educate themselves on their menstrual health is consistent with the key finding of an Australian-based survey that researched menstrual health literacy and management strategies in young women.^21^ This group found 50% of 4202 participants to engage with self-management of menstrual symptoms, including research on the Internet, instead of seeking medical advice, even in severe cases. This huge proportion of young women shows there is a great demand from women to want to understand their menstrual cycle, but they may feel unsupported by medical professionals while lacking the vital education regarding the understanding, management and awareness of menstrual health, something that is being addressed by the International Fertility Education Initiative.^22^ Feelings of confusion and being intrigued by variations in their menstrual cycle was also a key theme brought up by respondents in later qualitative analysis, further exemplifying the want and need for increased menstrual education.

An interesting finding was that when respondents were asked to select the phrase that best describes their attitude towards entering data into their app, while a few selected ‘I set an alarm/reminder to enter data as otherwise I would forget’, and ‘I only enter data when I need to know when my period will be (e.g. holiday)’, there was an opposing almost 50/50 split between the majority of respondents attitudes. One majority, 43.0%, of respondents described entering their data to be part of their routine. Meanwhile, the other majority, 37.0%, selected ‘I often forget to enter data as it is not a priority’.

This study found 83.0% of respondents to state they have no concerns regarding the privacy of their data when using a period tracker. When further questioned, most described how they had either never considered this, did not feel they entered data that could be used against them or that companies’ access to personal data is expected these days. This overall relaxed approach to data privacy agrees with findings from a mixed-methods study that aimed to reveal consumers' attitudes towards privacy and security when using health apps.^23^ This group found that these attitudes are highly contextualized and dependent on what the app is designed to be used for. A more recent study also found users are less concerned about privacy if they feel the benefits of the app were worth it.^24^ It could therefore be possible for the users to feel that the benefits of using their period tracker outweigh any data privacy concerns and they are not worried about the use of any data they do enter. The contextualized nature of privacy concerns is consistent with the findings from Proudfoot et al.,^25^ who found privacy concerns were more likely to be associated with the use of mental health apps. This is of particular importance given the results of a recent study that found 88% of 158 mental health apps collect users’ data and 49% of these share users’ data with third parties.^26^ Although specific to mental health apps, this could suggest that health apps as a whole may not be clear enough as to what they use users’ data for. The lack of privacy concerns in this present study could therefore be due to a lack of user concern regarding the data they are entering, combined with a lack of honesty from tracking apps regarding their data protection policies. An area for further research could investigate the period tracker’s privacy notices, to confirm users are not being falsely reassured through a lack of transparency.

### Ovulation prediction

As we have shown, ovulation prediction from a period tracker that only uses dates is highly inaccurate and to predict ovulation, an FABM needs to be measured;^1,4,9^ 29.7% stated the best thing about using a period tracker is ‘To know when I’m ovulating’ and 10.9% said they avoid having sex on the fertile days predicted by the app. These results highlight the trust women have in their period trackers and illustrate the potential responsibility these apps could have regarding unplanned pregnancies.

It is therefore recommended that period tracker apps stop predicting ovulation dates without physiological evidence inputted by the individual. In parallel, women should be made aware of other ovulation tracking methods, such as urinary ovulation detection kits, which work by measuring the luteinizing hormone surge which happens about 40 h before ovulation, to increase knowledge, and consequently empowerment, of individuals’ own menstrual cycles.

### The effect on women’s feelings and behaviours

The qualitative analysis of this survey provided an in-depth analysis of women’s experiences of using period tracker apps. This portrayed a sense of vulnerability as respondents wrote extensively and passionately about how their period tracker either affects or does not affect them. Overall, it appears as though users either rely on their tracker heavily and that it has a strong effect on their feelings and behaviours, or that users have learned to not rely solely on an app to predict period start dates and are no longer affected by its predictions. The range of words respondents selected when asked ‘How does/did using a period tracker app make you feel?’ reflects further this range in feelings and behaviours. Although the most frequently selected feelings were positive and those that would be expected from using a period tracker, such as ‘Prepared’ and ‘In control’, feelings such as ‘Frustrated’ and ‘Anxious’ were more commonly selected than ‘Confident’ and ‘Competent’. Feeling ‘Indifferent’ was also more frequently selected than other positive feelings such as ‘Knowledgeable’, ‘Empowered’ and ‘At ease’. Despite this majority selecting to feel positively towards using their tracker, it is likely respondents are more likely to feel this way since they have been interested in downloading and using one. There is still a proportion, a potentially a much greater proportion who were not included in the sample for this reason, who feel as though using a period tracker app is, or would, not be beneficial to them. An area of future work could combine previously mentioned further research regarding data protection policies to uncover whether if users were more aware of the apps intended use and data protection policies, would their feelings towards using their tracker change.

It emerged that only 6.7% of respondents stated their app always gets the date of their period correct and that it was more likely for respondents’ periods to start later than the apps predicted start date. A reason for this could be, as mentioned by Bull et al.^1^ and Faust et al.,^16^ that women tend to have a cycle longer than the traditionally and clinically cited 28-day cycle.^27^ Furthermore, Grieger and Norman^17^ found only 0.17% of women to have a short cycle (<21 days), compared to 8.60% to have a long cycle (>35 days). When partnered with a finding from Levy and Romo-Avilés,^6^ which described how app-facilitated period tracking promotes the idea that completely regular menstrual cycles are automatically associated with good health, this inaccuracy could be causing unnecessary worry. Although it is important for women to recognize that certain, and normally large, irregularities can be signs of pathology, only when it is possible to take a patient case as whole can this be confirmed. Irregular menstrual cycles are common, not always pathological, and caused by a multitude of other factors. This is consistent with previous work investigating variation in menstrual cycle length, where 29.3% of the 2865 women showed >14 days variation.^28^ This study concluded by opposing the idea that intra-individual variation of >5 days should be associated with the disease. With further research into this field, it may be possible to increase the understanding of menstrual regularities and the irregularities associated with pathology in order to refine the definition of a ‘normal’ menstrual cycle length. Once this greater understanding is achieved, it could then be possible for period tracker apps to be more in tune with their varied users.

The need for this research is further demanded when comparing the overall feelings described by respondents when their period is earlier than predicted, versus when their period is later than predicted. In general, when a period was late, it appeared women were more likely to be negatively affected, either by feelings of anxiety, disappointment, upset and frustration. However, an earlier period meant respondents were more likely to describe feeling unprepared or it being an inconvenience and slightly frustrating. This is consistent with the idea that the strongest feelings in either scenario were centred around pregnancy; a thought much more likely to cross users’ minds when their period is late.

### The effect of a period earlier than predicted

Analysis of the question ‘How did it make you feel when your period started earlier than the app predicted?’ produced four key themes: feeling unaffected, being frustrated and unprepared, feeling anxious and stressed, and being confused and intrigued. As previously discussed, although the majority of respondents felt either unaffected or frustrated at being unable to be prepared, a few described more concerning feelings of being frustrated at their own period and blaming their body. Not only does this highlight the trust some users have in their period tracker app but also, on a wider scale, illustrates the negative effects modern technology can have on users’ mental health and body confidence. One study investigated the different uses of technology and the relationship with physical and mental well-being in 244 undergraduate students.^29^ They found dependence on technology and devices, which users of period tracker apps are susceptible to, was associated with body image dissatisfaction, depression, and anxiety. The ease of being able to complete so many day-to-day tasks on a smartphone, including tracking periods, could mean our society has become too reliant on technology for the validation of our lifestyles and bodies.

### The effect of a period later than predicted

Analysis of the question ‘How did it make you feel when your period started later than the app predicted?’, produced 6 key themes: anxious and concerned about being pregnant, disappointed about not being pregnant, seeking advice and informing HCPs, thoughts about the menopause, feeling unaffected and being better prepared. The most frequently cited was feeling anxious and concerned about being pregnant, highlighting the sensitivity and importance of pregnancy to women, whether planned or unplanned. For some respondents, there were thoughts and concerns regarding the onset of menopause when their period was late. Although in this scenario, it is helpful to pick up a delay in the period start date, it further highlights the sensitive topics of gynaecological health these apps are dealing with and how essential it is apps are clear on the extent they are able to understand and predict individuals’ cycles.

Respondents’ comments about the menopause demonstrate how app-facilitated period tracking has the potential to be an insightful and constructive habit, as early peri-menopause is defined by increased variation in menstrual cycle length.^30^ The ability to monitor and then recognize any changes or extremes in period dates and symptoms allows users to get to know their usual cycle characteristics. If predictions were more accurate, any variation from the predicted dates could be taken more seriously. This would not only help women to recognize when they might be entering the peri-menopause, but also in the diagnosis of reproductive health conditions that remain a challenge to diagnose, such as poly-cystic ovarian syndrome (PCOS).^31^ Therefore, the ability for patients to recognize any extremes in cycle length and the increase in accurate information patients are able to provide to their HCP through data from their period tracker may improve the rates of efficient and beneficial diagnoses.

Certain respondents who were on contraception appeared less anxious and worried when their period was later than predicted. Not only does this reflect the need for users to be educated and apps to be clearer that they cannot be used as a reliable form of contraception, but also shows the importance of access to effective contraception. The WHO describes how access to preferred contraceptive methods encompasses more than just health benefits, with increased empowerment and education for women – an overall aim of this present study.^32^

### Limitations of the study

As with any survey, this study is limited to the responses of the people who completed it. Promoting surveys on social media gives a bias to the social media followers of the person advertising the survey. The majority of women were from the United Kingdom but not all were.

## Conclusion

This study shows that period tracker apps have the potential to have a positive impact on women’s lives, but only if users are aware of the limitations of app-facilitated period tracking and are able to understand the signs, symptoms and variations of their individual menstrual cycle and reproductive health. This study also demonstrates the responsibility period tracker apps have and the trust users have in them, as the impact of inaccurate predictions extends into other aspects of users’ health, including their mental health. Period tracker apps must be more transparent with the extent of their ability to accurately predict menstrual cycle dates, as well as their data protection policies. This study calls for period tracker app companies to update their apps to be clearer to their users about their intended use and capabilities. Alongside this, the increased regulation of such apps that are available to download freely is essential. Despite popular publications, such as Your Fertile Years, initiating unprecedented discussions, this study highlights a need for increased research into menstrual health. This will not only warrant evidence-based predictions by period trackers, but also ensure women are adequately educated about this consuming, sensitive, and constant aspect of their reproductive lives. On a global scale, this demonstrates the wider need for increased education and research in women’s health to address the inequalities in biomedicine.

## Supplemental Material

sj-doc-1-whe-10.1177_17455057221095246 – Supplemental material for A survey of women’s experiences of using period tracker applications: Attitudes, ovulation prediction and how the accuracy of the app in predicting period start dates affects their feelings and behaviours Click here for additional data file. Supplemental material, sj-doc-1-whe-10.1177_17455057221095246 for A survey of women’s experiences of using period tracker applications: Attitudes, ovulation prediction and how the accuracy of the app in predicting period start dates affects their feelings and behaviours by Anna Broad, Rina Biswakarma and Joyce C Harper in Women’s Health
